# Photosynthetic Performance of Green Alder (*Alnus alnobetula*) in Experimental Drought

**DOI:** 10.3390/plants15162537

**Published:** 2026-08-21

**Authors:** Andreas Gruber, Gerhard Wieser, Walter Oberhuber

**Affiliations:** Department of Botany, University Innsbruck, Sternwartestrasse 15, 6020 Innsbruck, Austriawalter.oberhuber@uibk.ac.at (W.O.)

**Keywords:** acclimation, climate change, drought stress response, photosynthetic quantum yield, plant vitality, stomatal conductance

## Abstract

Over the past decades, green alder (*Alnus alnobetula*) has spread rapidly across the Alps, expanding from moist, north-facing slopes into areas with limited water availability. Despite its growing ecological relevance in alpine environments, the species’ physiological limits under drought are largely unknown. To evaluate the species’ drought tolerance, we conducted a greenhouse experiment in which juvenile plants were subjected to drought periods of different durations, while monitoring stomatal conductance (gs) and quantum yields of photosystem II. Once the soil water potential fell below the wilting point (Θ*_WP_*), gs declined to less than 25% of the control level. After re-irrigation, gs remained reduced for several weeks, indicating a post-drought legacy effect. Under prolonged drought, the maximum quantum yield of photosystem II (Fv/Fm) declined to 20% of control values. The correlation between effective quantum yield and radiation proved a useful indicator for moderate drought stress. During recurrent drought, plants showed an acclimation to dry conditions, and both gs and Fv/Fm declined significantly (*p* < 0.01) at moderate drought, indicating a short-term, drought-induced adjustment in stomatal regulation. Although green alder showed signs of acclimation under repeated drought, recurrent severe drought led to high mortality in juvenile plants, indicating vulnerability at drought-prone sites.

## 1. Introduction

Climate change is projected to exert considerable pressures on mountain ecosystems [1,2,3]. However, in the coming decades, land-use change and pasture abandonment may be as important as rising temperatures in shaping treeline ecotones [4]. Recent studies have highlighted shrub encroachment in alpine pastures [5] and, in particular, green alder (*Alnus alnobetula*; syn. *A. viridis*) has widely invaded abandoned grasslands across the Alps [6,7,8]. Once restricted to well-drained, north-facing slopes and gullies [9], this species is now expanding into moderately moist alpine grasslands and even dry, south-facing sites [10]. Facilitated by clonal growth and nitrogen-fixing actinorhizal symbiosis, green alder forms dense thickets with canopies reaching four meters in height. As a result, nutrient-poor, species-rich grassland may gradually be transformed into nitrogen-rich shrubland with low diversity [8].

Alpine regions are experiencing warming that far exceeds the global average [11]. Studies suggest that rising temperatures may enhance growth in arctic and alpine regions [12]. However, reduced snow cover in winter and higher evapotranspiration rates during the growing season may make water availability a key limiting factor in arctic and alpine regions within the coming decades [12,13]. Since the 1990s, drought has emerged as a main driver of alpine shrub growth [14], and many alpine deciduous species are highly drought-sensitive [15]. Green alder also appears to be susceptible to drought stress, as indicated by premature leaf wilting under dry conditions [16] and significantly reduced height and radial growth at drier sites [10]. Green alder is considered an anisohydric species [17], keeping its stomata open even under high vapor pressure deficits [18,19,20], a strategy that can be risky as droughts become more frequent [21,22]. Green alder canopy transpiration and water use have been investigated in recent years [19,23,24], but studies on the species’ ability to cope with drought stress are still missing.

Drought-induced stomatal closure reduces water loss, but increases the risk of photo-oxidative stress and photoinhibition [25]. Chlorophyll fluorescence is widely used to assess drought effects in woody plants (e.g., [26,27]), with dark-adapted Fv/Fm providing an indicator of stress and damage to the photosynthetic apparatus (e.g., [28,29,30]). On the other hand, light-adapted parameters such as the effective quantum yield of photosystem II (Φ_PSII_) are used to detect protective downregulation of PSII photochemistry under moderate drought stress [31] and to assess photosynthetic acclimation [32].

The duration, intensity, and frequency of drought are critical, as they determine the extent of potential damage to both the photosynthetic apparatus and plant hydraulics [33]. During a severe drought, an increase in the xylem tension promotes cavitation events in the xylem [34], which can spread through the xylem network under prolonged tension [35]. Concurrently, drought triggers a shift in the biochemical control of the photosystems where progressive metabolic downregulation leads to photoinhibition [36,37] and, under prolonged stress, to the degradation of photosynthetic protein complexes [38]. Post-drought legacy effects [22,39], arising from damage to the hydraulic system, the photosynthetic apparatus or the root system [40], can have long-term impact on water and carbon balance [39,41].

To our knowledge, no study has examined the stomatal conductance (gs) and photosynthetic quantum yield of green alder under limited soil water availability. Therefore, the objective of this study was to assess the impacts of short, prolonged, and recurrent soil drought on stomatal conductance, maximum and effective quantum yield, and the vitality of green alder. We hypothesized that (i) drought will reduce stomatal conductance and quantum yield; (ii) drought will induce legacy effects and adjustments in stomatal regulation; and (iii) recurrent drought will cause high mortality in juvenile plants.

## 2. Results

### 2.1. Environmental Conditions

**Figure 1:**
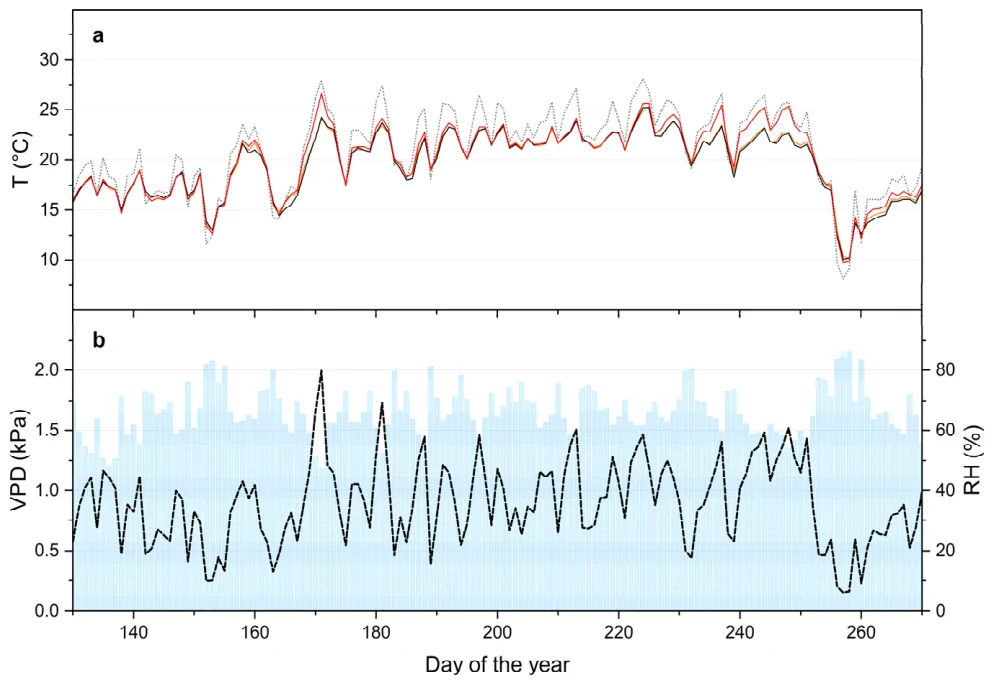
(**a**) Daily mean air temperatures (grey dotted line) and soil temperatures in the three experimental groups (C: black line, D1: orange line and D2: red line); (**b**) VPD (black dashed line) and relative air humidity (blue bars) over the course of the experiment.

**Figure 2:**
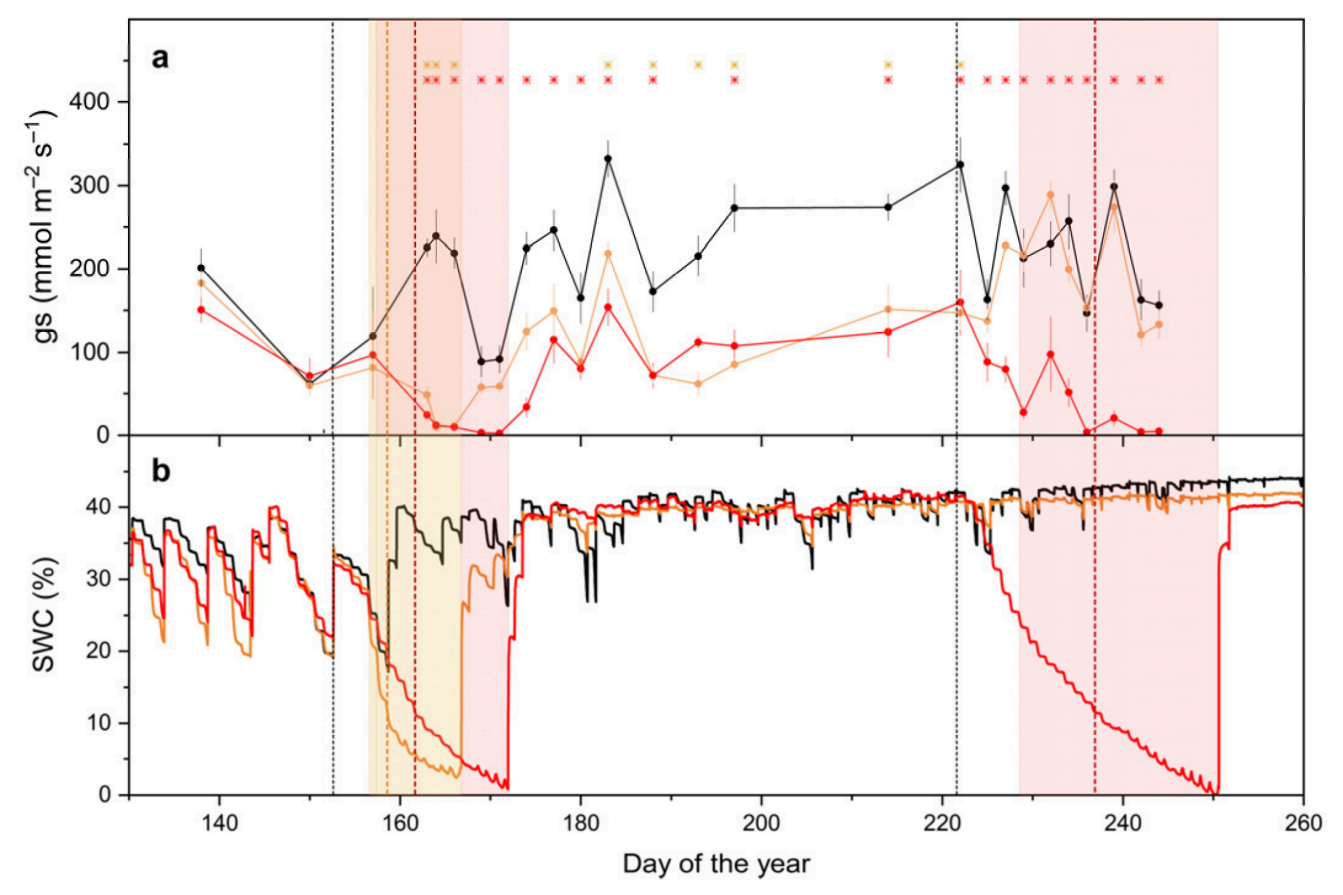
(**a**) Stomatal conductance (gs) at noon and (**b**) volumetric soil water content (SWC) in the three experimental groups; (C: black symbols and lines, D1: orange symbols and lines, and D2: red symbols and lines). The semi-transparent orange (D1) and red (D2) areas denote periods of limited water availability (soil water potentials < *Θ_la_*). black dotted vertical lines indicate the last watering before the onset of drought periods; orange (D1) and red (D2) dotted vertical lines mark *Θ_WP_*; orange and red asterisks denote gs values that are significantly different from the control (C).

During the experiment (early May–late September 2024), the mean daily minimum and maximum air temperatures in the greenhouse were 8.1 °C and 28.1 °C, respectively (Figure 1a). The mean daily relative humidity (RH) ranged from 45 to 86%, and the mean daily vapor pressure deficit (VPD) ranged from 0.14 to 1.99 kPa (Figure 1b). Before the first experimental drought, soil temperatures in the drought-stressed groups were not significantly different from those in C. Following experimental drought, the soil temperatures in D1 and D2 were slightly higher than in C (<0.5 °C), most likely due to reduced shading after leaf loss. Differences were more pronounced during experimental drought (up to 3 °C), most likely due to reduced evapotranspiration from drier soils, with additional contributions from leaf loss late in the drought. Elevated soil temperatures can influence stomatal conductance, but the effect probably was minor. No significant differences in SWC were observed until the start of the drought experiments (Figure 2a,b). Withholding water caused a continuous decline in SWC down to values < 5% in both the D1 and the D2 treatment. After re-irrigation, the soil water availability quickly recovered to values comparable to those of the control group (Figure 2b).

### 2.2. Stomatal Conductance During the Drought Experiments

**Figure 3:**
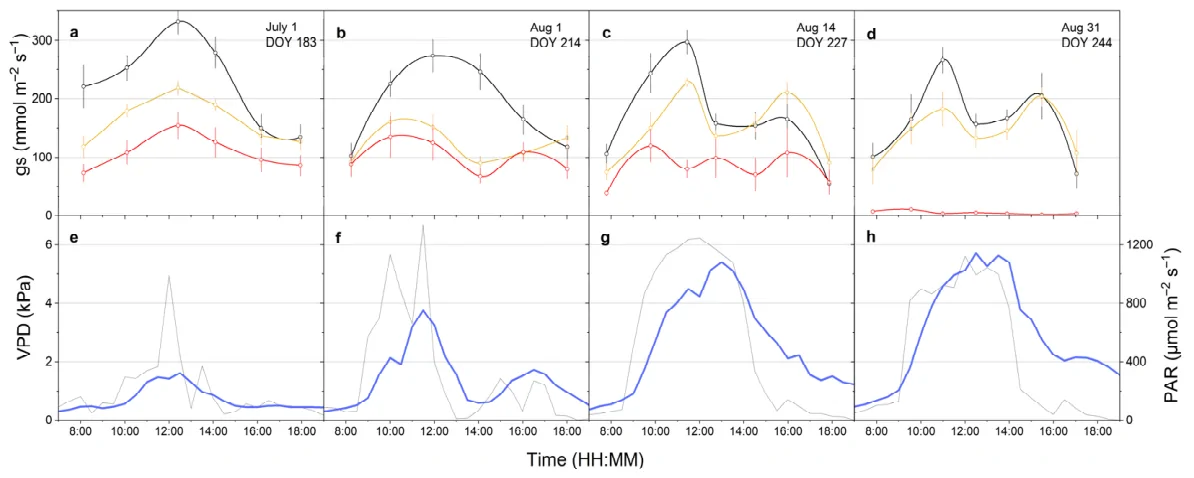
(**a**–**d**) Diurnal courses of stomatal conductance (gs), (C: black symbols and lines, D1: orange symbols and lines, and D2: red symbols and lines); (**e**–**h**) VPD (blue line) and PAR (grey line).

The gs of the experimental plants followed a diurnal pattern (Figure 3) but also varied markedly with environmental conditions. We used midday gs to validate drought effects, as the patterns were most pronounced at this time. During the season, the mean midday gs in controls varied from 62.0 ± 12.2 to 332.3 ± 22.1 mmolm^−2^s^−1^. Before the calculated threshold of limited water availability (*Θ_la_*: pf= 2.76; SWC= 24.2%) was reached in the first drought experiment, there were no significant differences in gs among the three groups (Figure 2a). However, when soil water potentials had fallen below *Θ_WP_* (*Θ_WP_*: pf= 4.2; SWC= 11.6%), the gs was significantly lower in both drought-exposed groups than in C. Eight and seven days after *Θ_la_* was reached in D1 and D2, the gs at noon was close to zero in both D1 and D2 (10.0 ± 2.7 and 12.2 ± 3.9 mmolm^−2^s^−1^, respectively).

After re-watering, the gs in D1 recovered rapidly and was no longer significantly different from C two days later. In D2, prolonged drought resulted in a longer period with gs close to zero, and gs was still significantly reduced 16 days after re-irrigation (72.3 ± 14.9 mmolm^−2^s^−1^) compared to C (172.6 ± 24.3 mmolm^−2^s^−1^) (Figure 2a). Gs in both drought-stressed groups remained depressed long after the drought experiment. In D1, gs was significantly reduced over the following eight weeks, and in D2 it stayed significantly below control levels on all but one measuring day up to the start of the second drought experiment.

During the second experimental drought period, gs responded quite sensitively to the reduced water availability. One day after *Θ_la_* was reached, the gs had fallen to 27.4 ± 49.2 mmolm^−2^s^−1^ and remained low until, seven days later, it was near zero (3.6 ± 4.0 mmolm^−2^s^−1^). At this time, the soil water potential was still above the wilting point (*Θ_WP_*). Gs remained close to zero until measurements were stopped 22 days after *Θ_la_* was reached, when no further leaf-level measurements were possible. A repeated-measures analysis indicated significant differences among the three groups over the course of the season (all post hoc tests *p* ≤ 0.001).

### 2.3. Diurnal Courses of Stomatal Conductance

The diurnal courses of gs varied from day to day depending on weather conditions. However, effects from drought treatment were evident. We selected representative days to highlight the effects of VPD and PAR on the diurnal course of gs. After the first drought treatment on July 1 (DOY 183), gs was significantly reduced in both D1 and D2 compared to C (*p*-values in Table S2; test statistics in Table S3) (Figure 3a). However, with PAR and VPD being rather low on that day, all three groups showed similar bell-shaped diurnal gs patterns. One month later, on August 1, with higher radiation and VPD, the gs of C was still rather high and showed a bell-shaped pattern (at noon 273.7 ± 27.6 mmol m^−2^ s^−1^), where the gs in D1 and D2 was significantly reduced over the course of the day. On August 14, at the beginning of the second drought experiment, gs in D2 was already significantly reduced. However, due to high PAR and VPD, C and D1 showed a distinct midday depression. This pattern was confirmed on August 31, with the gs in D2 being near zero during the whole day due to drought, while C and D1 showed a distinct midday depression driven by high VPD.

### 2.4. Leaf Water Potentials During the Drought Experiment

During the first experimental drought, Ψ at 8:00 a.m. in D1 and D2 was significantly reduced when soil water potentials fell below *Θ_WP_* (Figure S2). In D1, Ψ recovered within three days after re-irrigation and stayed at the control level until the end of this measuring campaign. Prolonged drought further reduced Ψ in D2 to −2.15 ± 0.49 MPa, and water potentials were still significantly different 11 days after re-irrigation (*p* = 0.037). In the second drought period, Ψ in D2 was significantly reduced (*p* = 0.028) three days after *Θ_la_* was reached and four days before the soil water potential had fallen to *Θ_WP_*. The ongoing drought reduced Ψ in D2 up to −2.22 ± 0.27 MPa.

During the first drought, Ψ at 3:00 p.m. in D1 and D2 was significantly reduced when the soil water potentials fell below *Θ_WP_*. While Ψ recovered quickly in D1 after re-irrigation, Ψ in D2 reached a minimum of −1.70 ± 0.43 MPa and remained significantly reduced until the end of this measuring campaign. During the second drought period, Ψ at 3:00 p.m. in D2 was not significantly reduced until 8 days after *Θ_WP_*.

### 2.5. Maximum Quantum Yield of Photosystem II

**Figure 4:**
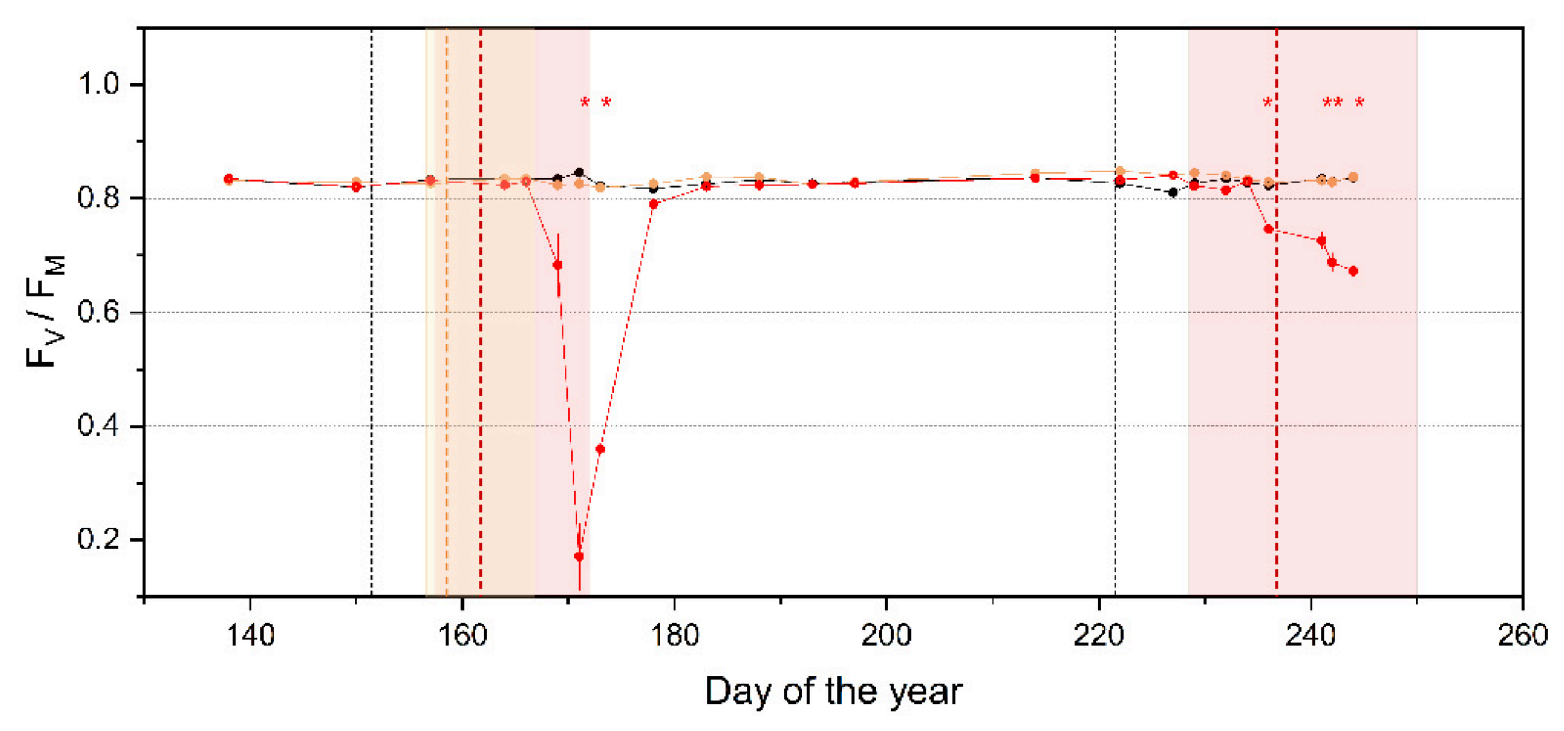
Maximum quantum yield of PSII (Fv/Fm) during the drought experiment in the three experimental groups. (C: black symbols and lines, D1: orange symbols and lines, and D2: red symbols and lines). The semi-transparent orange (D1) and red (D2) areas denote periods of limited water availability (soil water potentials < *Θ_la_*); black dotted vertical lines indicate the last watering before the onset of drought periods; orange (D1) and red (D2) dotted vertical lines indicate *Θ_WP_*; red Asterisks denote values in D2 that are significantly different from the control (C).

Fv/Fm was quite stable in non-drought-stressed samples throughout the season (0.830 ± 0.007). During the first experimental drought period, 10 days below *Θ_la_* did not significantly reduce Fv/Fm in D1. However, in D2, one sample showed a clear decline in Fv/Fm after 12 days below *Θ_la_*, whereas the others remained at the control level (Figure 4). Prolonged drought, with an additional two days below *Θ_WP_*, resulted in a significant reduction in Fv/Fm to 0.171 ± 0.059 across all samples (*p*-values in Table S2; test statistics in Table S3). Two days after rewatering, Fv/Fm in D2 was still significantly lower than in C, with half of the samples showing substantial recovery, whereas three samples had values close to zero four days later.

During the second experimental drought, Fv/Fm showed an early significant decline (0.746 ± 0.006; *p* = 0.003) one day before *Θ_WP_* was reached, with nine of ten measured leaves exhibiting lower values. Eight days later, at the final measurement, Fv/Fm reached a minimum of 0.673 ± 0.027.

### 2.6. Effective Quantum Yield of Photosystem II

**Figure 5:**
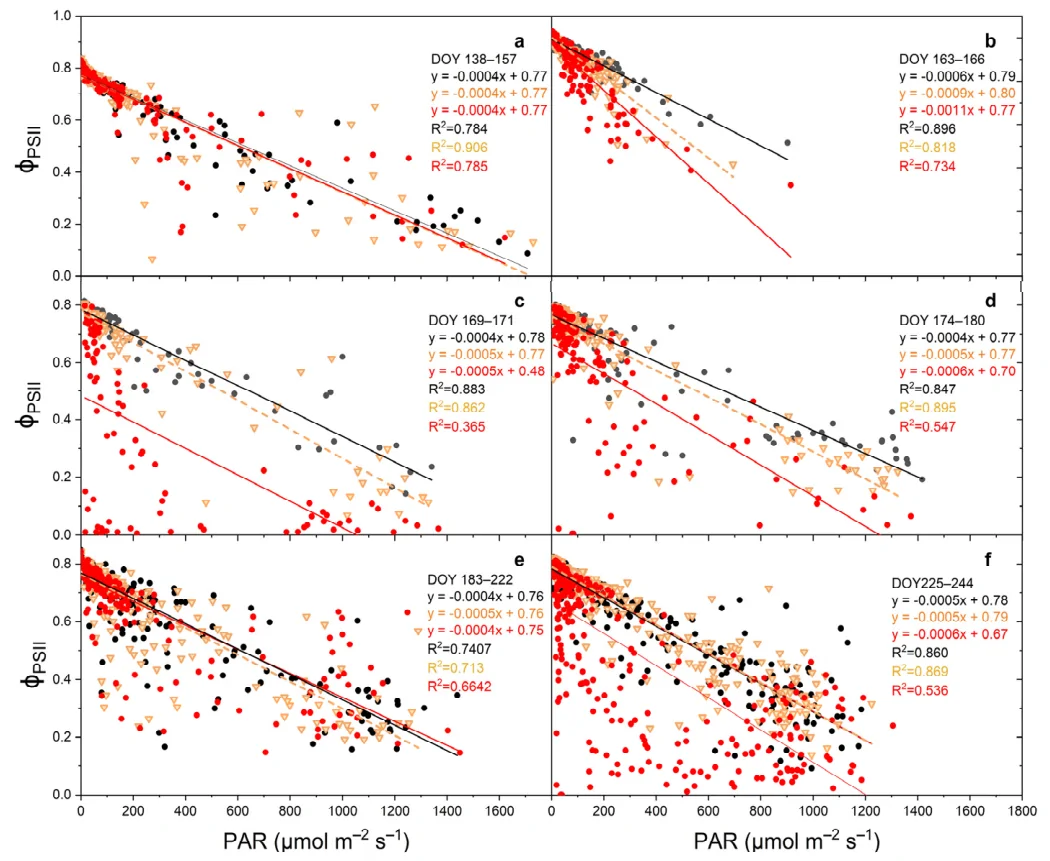
Effective quantum yield of PSII (Φ_PSII_) versus photosynthetically active radiation (PAR) (C: black symbols and lines, D1: orange symbols and lines, and D2: red symbols and lines). (**a**) Before first experimental drought, (**b**) during first drought period, (**c**) after re-irrigation D1 and ongoing drought in D2, (**d**,**e**) after re-irrigation of D1 and D2, (**f**) during second drought period. Lines are fitted linear regressions.

Φ_PSII_ is sensitive to changing light conditions, making it difficult to identify a stress response without taking radiation into account. Therefore, we examined the correlation between Φ_PSII_ and PAR to assess the response to drought stress at different stages of the experiment. Prior to the drought treatments, the regression slope and y-intercept did not differ significantly among the three groups, and R^2^ was also high in all groups (≥0.784, Figure 5a). During the first drought period (Figure 5b), the slope of the linear regression changed significantly in D1 and D2 (*p* = 0.0013 and *p* < 0.001, respectively), whereas the intercepts showed only minor changes. Prolonged drought in D2 (Figure 5c) led to a significant decrease in the intercept (*p* < 0.001), along with a decrease in R^2^. Following the reirrigation of all plants, the linear regressions for D1 and D2 remained significantly different from C (*p* = 0.028 and *p* < 0.001, respectively) (Figure 5d). From early July until the start of the second drought period, PSII had recovered, and no significant differences were observed in the linear regressions (Figure 5e). During the second drought period in D2, the linear regression y-intercept was significantly reduced (*p* < 0.001), along with a decrease in R^2^ (Figure 5f).

### 2.7. Leaf Loss Due to Drought Stress and Resprouting in Spring

Experimental drought caused wilting and leaf loss. During the first drought period, 10 days below *Θ_la_* resulted in about 25% of the leaves wilting in D1, while plants in D2 lost 75% of their leaves after 15 days. However, about 6 days after the reirrigation of D2, the plants of both groups started to grow new leaves. The new leaves replaced about 20% of the losses in D1 and D2. During the second experimental drought period, initial leaf wilting was observed on the day *Θ_WP_* was reached, following 8 days with soil water potentials below *Θ_la_*. Twelve days later, leaf-level measurements were no longer possible as the remaining leaves were severely damaged, and the experiment was stopped shortly after. However, by mid-October, 8 of 15 plants had sprouted some new foliage. The following spring, budbreak began on 2 April and 4 April in C and D1, respectively. In D2, budbreak was slightly delayed to 7 April. In C and D1, all plants sprouted new leaves. In contrast, only 5 of 15 plants in D2 were able to sprout leaves, and only two plants showed shoot and radial growth. Ultimately, 13 of 15 plants in D2 did not recover in the following spring. Thus, recurrent prolonged drought significantly reduced the survival of the juvenile green alder plants (*p* < 0.001).

## 3. Discussion

Despite its growing ecological relevance in alpine environments, the physiological limits of *Alnus alnobetula* under drought conditions are not well understood. There is evidence that reduced water availability limits the productivity of this pioneer species [10,23], and that drought may become a limiting factor for its spreading in a future warmer climate [12,16].

Gs varied considerably with environmental conditions, but values for non-stressed plants were comparable to those reported for alpine sites [19]. Because soil water availability strongly regulates stomatal conductance [42,43], the pronounced decline in gs was expected once the soil water potential dropped below *Θ_WP_*. Stomatal closure is the main short-term mechanism that limits transpiration losses and enables sufficient hydration during drought [43,44]. Green alder has been classified as anisohydric [19,20], meaning it does not strictly regulate leaf water potential via stomatal closure, so transpiration can remain relatively high as soils dry [45]. However, plant responses to drought are complex, making simple categorization problematic [42,46]. Our results indicate that VPD is a primary driver of stomatal regulation in green alder. Under sufficient water availability and low VPD, gs shows a bell-shaped daily course (Figure 3a), whereas under high VPD, gs displays a distinct midday depression (Figure 3c,d) [47,48]. Stomatal closure keeps water potentials within a physiological range [45], accounting for the moderate Ψ values observed even when gs was constrained (Figure S2). Overall, the Ψ values measured in the greenhouse were comparable to those observed at natural treeline stands. On sunny days, the minimum Ψ at drier sites reached −1.41 ± 0.02 MPa, whereas at moist sites it reached −1.15 ± 0.03 MPa. The experimental drought significantly decreased Ψ, with the length and severity of drought having a significant impact. After the short drought in D1, Ψ rapidly returned to pre-drought levels following rewatering (Figure 2 and Figure S2), suggesting sufficient water transport via remaining intact xylem [26]. In contrast, the prolonged drought in D2, which significantly depressed Ψ for several days, may have required repair mechanisms dependent on carbon reserves [49,50].

Fv/Fm measured in dark-adapted leaves reflects the maximum or potential quantum yield of PSII and is used as an indicator for plant stress (e.g., [28,29,30]). In our study, non-stressed leaves showed values of 0.830 ± 0.007, which fall within the standard range reported for healthy leaves in many species (e.g., [51,52]), while reduced values are seen as signs of stress and photoinhibition. It is well established that PSII is highly resistant to water deficits. Consequently, deciduous species can maintain high Fv/Fm at low soil water potentials [27,30,52]. During the first experimental drought, Fv/Fm followed this pattern, with reductions in Fv/Fm occurring only when soil water potentials were below *Θ_WP_* for several days (Figure 4). The rapid recovery of Fv/Fm after rewatering suggests a reversible, protective downregulation of PSII rather than structural damage [27,29]. It has to be considered, however, that some leaves used for measurements wilted during drought and had to be replaced by neighboring leaves of the same twig, which might have influenced the observed recovery.

While Fv/Fm shows little response to moderate drought [27], Φ_PSII_ is sensitive to moderate changes in soil water availability [31]. Although the relationship between Φ_PSII_ and PAR is physiologically curvilinear (saturating), the slope and y-intercept from linear regressions of Φ_PSII_ against PAR are commonly used to evaluate plant responses to drought and other stressors [31,32,53,54]. In this context, a steeper (more negative) regression slope indicates a reduced ability of the plants to dissipate excess energy under stress, while the y-intercept provides a daytime estimate of Fv/Fm [31,32]. Our data confirm that Φ_PSII_ versus PAR regression can be used as a proxy to assess moderate drought stress in green alder (Figure 5).

As hypothesized, following the first experimental drought, gs in D1 and D2 remained significantly lower than in C for several weeks before returning to comparable levels. Long-term, drought-induced legacy effects have been described for green alder and for other species from moderately cool, moist environments following drought years [15,55]. They may result from drought-induced hydraulic damage, photooxidative damage, or root mortality [39,40,56] and can have a long-term impact on water and carbon balance [39,41]. We suggest that the legacy effect observed in gs reflects damage to the hydraulic pathway or root system that required several weeks to repair, consistent with the gs in D1 returning to control levels after about two months. On the other hand, Fv/Fm quickly recovered after re-irrigation, indicating a metabolic downregulation leading to photoinhibition [36,37], rather than long-term damage to PSII [29,30].

During the second drought period, gs had already declined under moderate drought. Gs was strongly reduced at *Θ_la_* and close to zero before *Θ_WP_* was reached (Figure 3g). This early restriction of gs indicates a short-term response or adjustment in stomatal control, triggered by the preceding drought [57], as hypothesized. It is well known that plants alter their metabolic pathways in response to drought, thereby protecting against stress-induced damage [58,59]. Plants have evolved mechanisms to memorize past stress and prime their responses, enabling faster or stronger reactions to subsequent stress [60,61]. The mechanisms of information storage and retrieval include epigenetic, transcriptional priming, primed protein conformations, and characteristic hormonal and metabolic signatures [60]. Primed responses to recurring drought, as observed in the second drought experiment, are potentially mediated by jasmonic acid (JA) and abscisic acid (ABA) interactions [62,63]. A possible primed response was also evident in Fv/Fm, which was already significantly reduced under comparatively moderate drought prior to reaching *Θ_WP_*, during the second experimental drought. Given that gs was getting close to zero at the same time, we assume that the early decline in Fv/Fm may be linked to a stress-induced or primed stomatal closure. Beyond restricting CO_2_ supply, stomatal closure also increases the risk of photo-oxidative stress [30,64] and induces an early transition to biochemical limitation, with RuBP regeneration, ATP synthesis, and electron transport constraining photosynthesis. Progressive metabolic downregulation leads to photoinhibition [36,37] and ultimately to the degradation of photosynthetic protein complexes [38]. The significantly smaller decline in Fv/Fm during the second drought, once soil water potential fell below *Θ_WP_*, suggests a drought-induced adjustment of photoprotective mechanisms and non-stomatal limitations of photosynthesis following the first drought period [65,66].

Prolonged drought led to significant leaf loss during our experiments. Leaf shedding due to drought has been reported for many tree species (e.g., [17,67]) and is frequently associated with non-reversible declines in hydraulic conductance [68]. Reducing the transpiring surface is an effective strategy for surviving dry periods and for subsequent recovery [65,69,70]. It has also been suggested that resprouting species are more resilient to drought stress than non-resprouters [71]. All plants in our experiment resprouted new leaves after the first drought. However, after the second drought, only about half of the plants were able to resprout next spring, and only 2 of 15 ultimately survived.

Although green alder showed signs of acclimation under repeated drought, recurrent severe drought caused massive leaf loss, followed by missing budbreak in spring; 85% of plants failed to resprout after winter dormancy. Although short-term responses and longer-term, drought-driven adjustments of hydraulic and root systems on drier sites [40,72] might support further expansion under dry conditions, our results point to a high vulnerability to water limitation. With climate change increasing the frequency of extreme events [73], green alder will face more frequent droughts, reducing its competitiveness and heightening its susceptibility to parasites and diseases [74,75]. Therefore, our findings indicate that green alder may not be able to spread under future drier conditions or at drought-prone sites, as often suggested [6,7,8]. Given the inherent constraints of greenhouse studies, additional field measurements are needed to independently validate extrapolation of these results to natural habitats.

## 4. Material and Methods

### 4.1. Experimental Setup and Drought Conditions

Three-year-old, approximately 1 m tall green alder plants of a local provenance, raised in a local nursery, were planted in five-liter pots in late summer the year before the experiment. For planting, a standard garden soil (42% compost, 21% leaf mold, 21% topsoil, 16% sand) was used (soil water retention curve for the used soil see Figure S1). About one month before budbreak in spring 2024, the plants were placed into a greenhouse in the botanical garden in Innsbruck (47.2679° N, 11.3786° E; 600 m a.s.l.). The greenhouse was equipped with an automatic ventilation system to prevent overheating during sunny periods in summer.

To maintain a sufficient water supply, all plants were watered at regular intervals up to field capacity. The initial weekly watering schedule caused fluctuations in soil moisture. Therefore, at the start of the drought experiments, we switched to watering every two days for all groups during non-drought periods. Given that green alder tolerates and grows well in wet soils [9], the plants were maintained at field capacity between drought experiments.

The juvenile plants were divided into three groups (15 plants each), and the experiments were started about one month after the leaves were fully developed. While plants in the control group (C) were continuously watered, group D1 was exposed to one short drought (10 days), while group D2 was exposed to two prolonged drought periods (15 and 22 days). From the measured soil water retention curve for the experimental soil (Figure S1), we calculated the soil water potential corresponding to the point of limited water availability for plants (*Θ_la_*) [76].

*Θ_la_* = *Θ_WP_* + *a(Θ_fc_ − Θ_WP_)* (1)

where *Θ_la_* is the point of limited water availability for plants, *Θ_fc_* is the field capacity, and *Θ_WP_* is the wilting point. *Θ_fc_* and *Θ_WP_* values were given by the HYPROP2 soil water potential measuring system (see Section 4.2).

Using these parameters, threshold values of volumetric soil water content (SWC) were derived from the retention curve calculated by the HYPROP2 system employing the van Genuchten model (Table S1). We continuously monitored SWC throughout the experiment to determine when *Θ_la_* and *Θ_WP_* were reached. Plants in D1 and D2 were subjected to soil water potential below *Θ_la_* for 10 and 15 days, respectively. As drying continued, the soil water potential fell below the wilting point (*Θ_WP_*) one and four days after *Θ_la_* was reached, in D1 and D2, respectively. After the first drought period, all plants were rewatered to recover from drought stress. While D1 was continuously watered until the end of the growing season, D2 experienced a second prolonged drought eight weeks later, with a soil water potential below *Θ_la_* for 22 days.

During a natural drought period, we observed leaf wilting up to complete defoliation at a dry treeline site [16]. Accordingly, we used leaf wilting as an operational criterion to fine-tune the duration of experimental drought, both to prevent premature lethal damage to test plants and as a gauge of the stress they experience.

In early autumn, all plants were transferred outdoors and overwintered in the botanical garden. In the following spring, resprouting was assessed for all individuals.

### 4.2. Measurements and Analyses

Air temperature and relative air humidity were continuously measured at two meters height, using a shielded S-THC-M002 Smart Sensor. SWC was continuously measured for four samples per experimental group using 10HS Soil Moisture Smart Sensors, and soil temperatures at 10 cm soil depth were measured with a 12-bit temperature Smart Sensor (all smart sensors, HOBO Dataloggers, Bourne, MA, USA) and three i-Buttons per group (Maxim integrated, San Jose, CA, USA). The environmental sensors were connected to HOBO USB Micro Stations (HOBO Dataloggers, Bourne, MA, USA).

A high-resolution soil water retention curve was obtained for five samples of the experimental soil using a HYPROP2 system (METER Group, Pullman, WA, USA), following the method described by Shokrani and Ghane [77]. The resulting retention data were then fitted to derive the parameters used in our analyses.

Because transpiration and photosynthesis are subject to diurnal rhythms, driven by regular changes in environmental parameters and endogenous regulation [78,79,80], gs and effective quantum yield of photosystem II (Φ_PSII_) were measured in diurnal cycles, at one-to-two-hour intervals, to gain detailed insights into green alder’s response to drought stress.

The diurnal courses of gs and the Φ_PSII_ were measured in situ in five plants per group, which were homogeneously distributed within each group. On each plant, a sun-exposed branch bearing approximately five leaves was marked, and the uppermost leaf was used for measurements throughout the experiment. If that leaf wilted beyond suitability for measurement, the next highest sun-exposed leaf on the same branch was used. The maximum quantum yield (Fv/Fm) was determined predawn in dark-adapted leaves. For these measurements, ten sun-exposed twigs were marked, and the uppermost leaf was used for measurements. On each measurement date, six to ten leaves were measured (six in non-drought phases and ten during drought). When leaves wilted, we followed the procedure described above. All measurements were performed with a Mini-PAM-II/Porometer (Heinz Walz GmbH, Effeltrich, Germany) using blue light illumination with a maximum wavelength of 474 nm and a standard modulation frequency of 15 Hz, with the saturation flash intensity set to 5000 μmol m^−2^ s^−1^. XLeaf temperature, relative air humidity (RH), and photosynthetically active radiation (PAR) for each measurement were assessed by sensors inside the leaf chamber.

During the experimental drought periods, leaf water potentials (Ψ) were measured using a M 600 pressure chamber (PMS Instrument Company, Albany, OR, USA). Measurements were taken at 8:00 a.m. and 3:00 p.m. on five leaves per group.

### 4.3. Statistical Analysis

Gs, Ψ and Fv/Fm at different measurement dates were tested for statistical differences between the experimental groups using Kruskal–Wallis tests, Dunn post hoc tests, and Bonferroni correction. Differences between groups over time were tested for significance with a repeated-measures analysis using Bonferroni correction. Correlations between Φ_PSII_ and photosynthetically active radiation (PAR) in D1 and D2 were tested for statistical difference from correlations of controls using covariance analysis (Ancova). Φ_PSII_ data were grouped according to PAR levels (group size 40 µmol m^−2^) prior to statistical analysis to compensate for large numbers of measurements at low irradiances. The difference in survival rates between groups was tested using Fisher’s exact test. All datasets were checked for normality and homogeneity of variance before conducting statistical analyses. Because multiplicity across the season was not corrected, between-group significance in gs and Fv/Fm should be interpreted with caution. Statistical analyses were performed using SPSS Statistics, version 29.0.2.0 (IBM, New York, NY, USA) and R Studio, version 2024.09.0 (RStudio Inc, RStudio PBC, Boston, MA, USA).

## Data Availability

The data underlying this article are available in Zenodo: https://doi.org/10.5281/zenodo.21410302.

## Supplementary Materials

The following supporting information can be downloaded at: https://www.mdpi.com/article/10.3390/plants15162537/s1. Figure S1: Soil water retention curve of the soil used in the experiment; Figure S2: Leaf-water-potentials measured in the three experimental groups; Table S1: Pf-values and volumetric soil water content of thresholds used for quantifying drought periods; Table S2: *P*-values from Kruskal Wallis tests comparing group C and groups D1 and D2 for gs and Fv/Fm across measurement dates; Table S3: Kruskal–Wallis and post-hoc pairwise test statistics.
