# Supplementary material for: Photosynthetic Performance of Green Alder (Alnus alnobetula) in Experimental Drought
Source: Plants (Basel). 2026 Aug 21;15(16):2537. doi: 10.3390/plants15162537 (PMC13516117; doi:10.3390/plants15162537)
Supplement: Supplementary file 1 [file plants-15-02537-s001.zip › plants-4475320-Table S1.pdf]

Table S1: Pf values and Volumetric soil water content (SWC) of thresholds used for quantifying drought periods

Threshold	pf	SWC (%)
Θ_{fc}	1.80	35.73
Θ_{la}	2.76	27.17
Θ_{WP}	4.20	11.56

Θ_{fc} and Θ_{WP} are given by the a HYPROP2 system and Θ_{la} is calculated using formula 1. SWC values were calculated by the HYPROP2 system using the Genuchten equation.
