# Supplementary material for: Photosynthetic Performance of Green Alder (Alnus alnobetula) in Experimental Drought
Source: Plants (Basel). 2026 Aug 21;15(16):2537. doi: 10.3390/plants15162537 (PMC13516117; doi:10.3390/plants15162537)
Supplement: Supplementary file 1 [file plants-15-02537-s001.zip › plants-4475320-Table S2.pdf]

Table S2: P-values from Kruskal Wallis tests comparing group C and groups D1 and D2 for gs and Fv/Fm across measurement dates.

	p-values gs		***p-values Fv/Fm***	
**DOY**	**C-D1**	**C-D2**	**C-D1**	**C-D2**
**138**	0.140	0.601	0.671	0.750
**150**	0.893	0.773	0.161	0.782
**157**	0.650	0.267	0.671	0.671
**163**	0.046	0.001	-	-
**164**	0.006	0.011	0.972	0.535
**166**	0.013	0.008	0.887	0.571
**169**	0.289	0.001	0.228	0.096
**171**	0.358	0.002	0.076	0.001
**174**	0.214	<0.000	0.999	0.018
**177**	0.127	0.008	0.395	0.288
**180**	0.059	0.017	-	-
**183**	0.040	0.001	0.386	0.397
**188**	0.020	0.006	0.665	0.400
**193**	0.001	0.066	0.217	0.180
**197**	0.000	0.001	0.750	0.750
**214**	0.048	0.007	0.663	0.563
**222**	0.015	0.023	0.077	0.437
**225**	0.572	0.048	-	-
**227**	0.150	<0.000	0.157	0.146
**229**	0.940	0.011	0.225	0.643
**232**	0.185	0.010	0.604	0.089
**234**	0.468	0.004	0.645	0.571
**236**	1.000	<0.000	0.512	0.003
**239**	0.621	0.004	-	-
**241**	-	-	0.932	0.015
**242**	0.423	0.002	1.000	0.001
**244**	0.525	0.003	0.671	0.002
