# Supplementary material for: Photosynthetic Performance of Green Alder (Alnus alnobetula) in Experimental Drought
Source: Plants (Basel). 2026 Aug 21;15(16):2537. doi: 10.3390/plants15162537 (PMC13516117; doi:10.3390/plants15162537)
Supplement: Supplementary file 1 [file plants-15-02537-s001.zip › plants-4475320-Table S3.pdf]

Table S3. Kruskal–Wallis test statistics (H) comparing groups C, D1, and D2 for gs and Fv/Fm across measurement dates (df = 2 in all cases), and post hoc pairwise test statistics (differences in mean ranks, ΔMR) for comparisons of group C with groups D1 and D2 for gs and Fv/Fm across measurement dates.

	H, gs	*ΔMR, gs*		*H, Fv/Fm*	*ΔMR, Fv/Fm*	
**DOY**	**C-D1-D2**	**C-D1**	**C-D2**	**C-D1-D2**	**C-D1**	**C-D2**
**138**	2.315	4.000	1.267	0,558	1,200	-0,900
**150**	0.477	0.333	-0.667	2,092	-3,700	0,775
**157**	1.286	-1.250	1.650	0,240	1,200	1,200
**163**	10.914	6.286	10.857	-	-	-
**164**	9.437	7.800	7.200	2,353	0,100	0,380
**166**	9.103	6.600	7.500	0,561	1,600	-0,400
**169**	10.500	3.000	9.000	2,961	3,400	4,700
**171**	10.220	2.600	8.800	12,590	5,000	10,000
**174**	11.580	4.200	11.100	8,734	1,200	7,886
**177**	6.020	4.600	7.633	3,428	-2,400	3,000
**180**	6.451	5.800	9.200	-	-	-
**183**	10.820	5.000	6.700	2,654	-2,600	-2,433
**188**	8.820	6.600	7.800	1,768	-1,300	2,417
**193**	12.020	9.800	5.200	3,273	3,900	3,700
**197**	13.538	15.000	13.200	0,406	0,900	0,900
**214**	1.814	5.600	7.600	4,829	-6,200	-2,800
**222**	5.804	6.800	6.000	3,140	2,800	-2,200
**225**	2.114	1.600	5.600	-	-	-
**227**	4.250	4.600	10.800	2,745	-4,000	-4,100
**229**	2.895	0.200	7.100	2,999	-3,400	1,300
**232**	4.436	-3.500	7.175	3,142	-3,600	1,200
**234**	4.900	2.000	7.500	1,059	-1,625	2,000
**236**	15.292	0.000	12.500	14,655	-2,528	10,722
**239**	7.814	1.400	8.200	-	-	-
**241**	-	-	-	12,008	0,750	11,114
**242**	5.727	2.250	8.000-	15,036	10,313	3,000
**244**	5.523	1.800	2.828	18,585	-1,500	11,063
